# Ebola Virus Disease: Experience and Decision Making for the First Patients outside of Africa

**DOI:** 10.1371/journal.pmed.1001857

**Published:** 2015-07-28

**Authors:** David S. Stephens, Bruce S. Ribner, Bryce D. Gartland, Nancye R. Feistritzer, Monica M. Farley, Christian P. Larsen, John T. Fox

**Affiliations:** Emory Serious Communicable Diseases Unit, Emory Ebola Task Force, Emory University School of Medicine, Emory Healthcare, the Robert W Woodruff Health Sciences Center and Emory University, Atlanta, Georgia, United States of America

## Abstract

David Stephens and colleagues describe their experience of treating patients with Ebola virus disease at Emory University in the United States.

**Summary Points**

- Ebola hemorrhagic fever, or Ebola virus disease (EVD), has emerged in the last year as a global threat and humanitarian disaster for the affected countries of West Africa and has also come to the United States (US) and Europe.
- The treatment of the first and three subsequent US patients outside of Africa at Emory University provided a number of challenges, as well as strategic and tactical lessons that included detailed planning and team work across multiple academic and health care units, emphasizing biosafety, the importance of institutional communications, addressing unanticipated challenges such as waste management, and the logistics of working closely with governmental agencies and outside collaborators.
- In providing effective care for individuals, the value of mobilizing a diverse health and academic community to work collaboratively to addressing a global threat is emphasized. This includes dissemination of best practice information; providing education and training about EVD; expansion of new knowledge about the clinical course, complications, and pathogenesis of EVD; the creation of new institutional forums, and engagement in the broader policy and equity issues of contagious health threats.

Ebola hemorrhagic fever, or Ebola virus disease (EVD), is caused by a highly contagious group of enveloped, single-stranded, negative-sense RNA viruses of the family Filoviridae. The disease has historically carried 53%–88% mortality [1–3]. In December 2013, what has now become the largest and most devastating Ebola outbreak ever recorded, caused by a new strain of the Zaire species (*Zaire ebolavirus*), began in Meliandou, Guinea, West Africa and soon enveloped neighboring Sierra Leone and Liberia, with cases also in Nigeria, Senegal, and Mali [1–5]. WHO (World Health Organization)/CDC (United States Centers for Disease Control and Prevention) reports 27,550 cases and 11,235 deaths in the three principal countries as of June 28, 2015 [6], which may be an underestimation of at least a factor of 2.

On August 2 and 5, 2014, two medical missionaries who developed EVD in late July while working at Eternal Love Winning Africa (ELWA) hospital in Liberia were evacuated from Monrovia by a specially designed air ambulance to Emory University Hospital (EUH) [7]. Until August 2, 2014, EVD had not been seen or treated in humans outside of Africa. The initial call for help for these missionaries came to our institution on July 30 and would mobilize multiple resources at Emory University, in particular those of the academic medical center, EUH, Emory Healthcare, and the Robert W. Woodruff Health Sciences Center, the faculty, nurses, staff, and leadership of these units, and our communications and other Emory University support teams. The successful treatment and discharge of these first two patients by August 21, 2014, was only the first chapter of the impact of this outbreak on Emory, the US, Europe, and the global community. At least 25 individuals have now been treated outside of Africa (US: 11, Europe: 14, with five deaths). The US, Europe, and the rest of the world can expect to continue to be touched by this epidemic and by future communicable disease outbreaks.

The strategic and tactical lessons and challenges presented by providing care for the first and subsequent US EVD patients at Emory University provide an opportunity to share lessons that may help others. Specific clinical and immunological features and diagnostic and detailed management issues of the patients at Emory are reported separately [7–11]. It is very important to acknowledge the tremendous inequity between EVD care available in our academic medical center versus sites in West Africa. This disparity, illustrated by this outbreak, will require much future work to address.

## Background

EUH is the flagship clinical care facility for Emory Healthcare, staffed by 1,221 Emory School of Medicine faculty physicians, with over 3,600 employees, 25,300 admissions, and 157,000 outpatient visits annually. The hospital has one of the highest case-mix indexes (a measure of complexity of illness treated) among the University Healthsystem Consortium (UHC)-ranked facilities. Emory had established strong programs in health care delivery, quality, infectious diseases (ID), infection control and biosafety, immunology, vaccines, and public health that were important to its ability to respond to this series of events.

**Fig 1:**
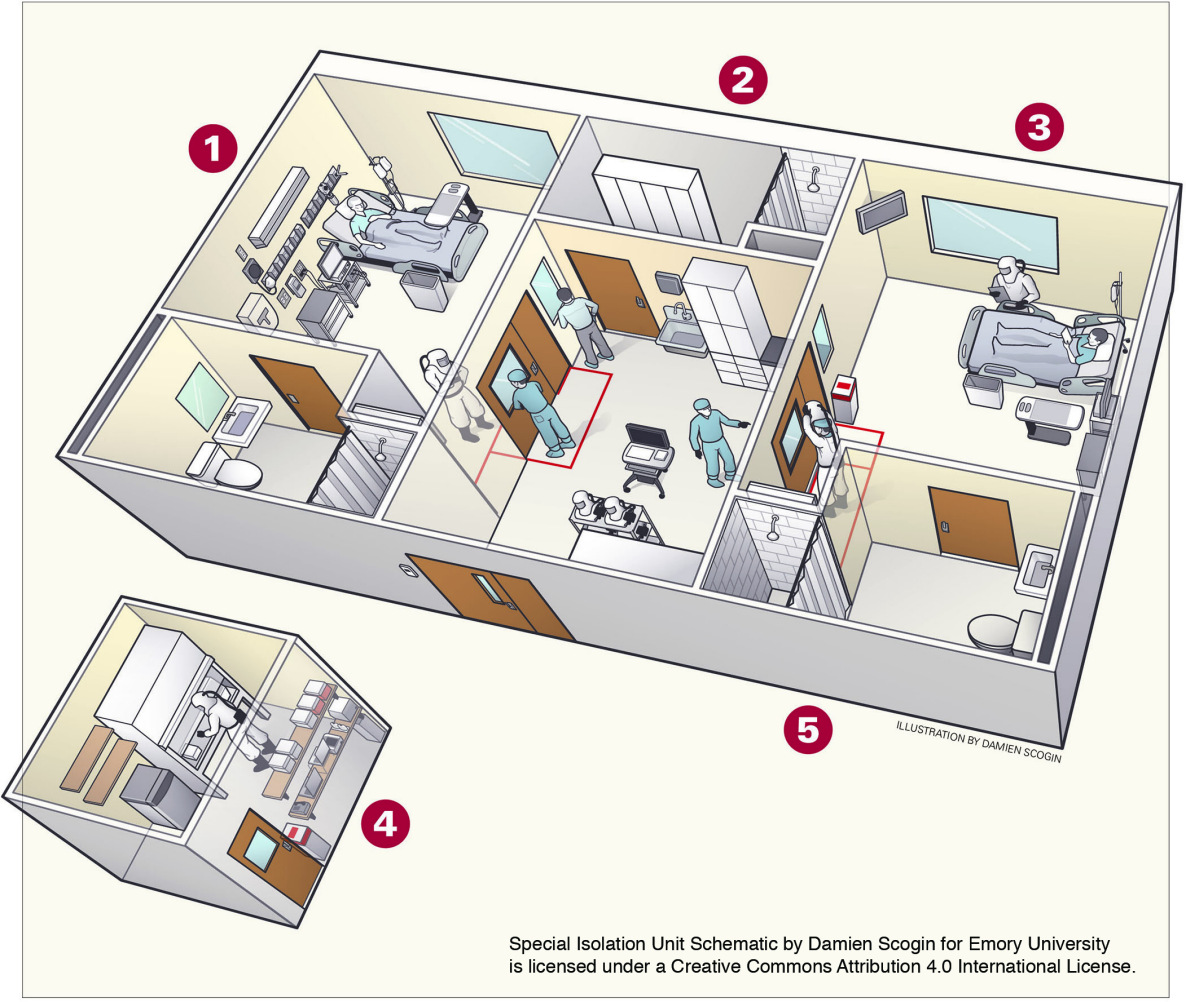
The SCDU. (1) The private patient rooms resemble intensive care unit (ICU) rooms, with adjustable beds, intravenous (IV) fluid drips and monitors. Procedures a patient could need, from mechanical ventilation to hemodialysis, can be performed in the unit. (2) Medical staff who are providing direct patient care use a locker room to change into full-body protective suits and masks, which shield them from blood and bodily fluids. (3) Family members are able to speak with patients through glass windows in the unit; patients have access to phones and laptop computers. The windows also allow observation of procedures and detection of contamination events. (4) A dedicated laboratory that has the capacity to perform blood counts, routine chemistries, blood gas measurements, urinalysis, and tests for a variety of infectious agents was built specifically for use with the isolation unit. (5) All liquid waste is disinfected and flushed, and disposable waste is autoclaved and incinerated. At the peak of the Ebola patient’s illness, up to 40 bags a day of medical waste were produced. *Image credit: Emory University; illustrator: Damien Scogin; licensed under a Creative Commons Attribution 4.0 International License*.

The Serious Communicable Diseases Unit (SCDU) (Fig 1, Box 1) of EUH is a special isolation unit. This unit was designed and constructed in 2002 following the 2001 anthrax bioterrorism event but was also created, given the global connectivity of Atlanta and its institutions, to address naturally acquired, highly communicable emerging infectious diseases such as EVD. The unit was built and set up in close collaboration with and with financial support from the adjacent CDC to provide care for CDC scientists and staff who have contracted highly communicable infectious diseases in laboratory settings or while traveling abroad.

**Box 1. The SCDU**

- The SCDU was designed (Fig 1) to provide an extraordinarily high level of clinical isolation with more enhanced capacities than are normally provided to isolate patients in many hospital settings. The unit has a special outside entrance for emergency use and is separated from patient care areas in order to not impact other clinical services. It was the first of three such high-level civilian facilities in the US.
- The SCDU was constructed (purposely to provide rapid isolation, security, flexibility, and access to special resources) within the EUH clinical research unit. The clinical research unit has been in existence for some 60 years and supports clinical research through the National Institutes of Health (NIH)-funded Clinical and Translational Science Awards (CTSA) program. The SCDU offers on-call, highly trained staff operating under standardized, ready-to-go protocols for providing care for patients infected with highly communicable contagious diseases.
- While not necessary for treatment of EVD, the isolation rooms feature negative pressure air handling, 20 air changes per hour, with laminar flow and high-efficiency particulate air (HEPA)-filtering completed before the air is 100% exhausted outside of the hospital (no recirculation). General and intensive care can be provided. The SCDU was activated on two previous occasions. The first was in 2003 for a CDC worker with concern for having been infected with severe acute respiratory syndrome (SARS) in Hong Kong, China. The second was in 2005 for a CDC employee in Angola with potential Marburg virus hemorrhagic fever. While unit activation has been infrequent, intensive training in this unit to maintain a state of readiness has been continuous for well over a decade, including detailed preparation for transport of patients and the training of nurses and physicians.
- A virtual tour of the SCDU and videos addressing many of the most frequently asked questions, together with access to all SCDU policies and standard operating procedures (SOPs), are available on the Emory education web site: www.emoryhealthcare.org/ebolaprep [11].

The ID program at EUH was built to provide expertise in hospital epidemiology, antimicrobial management, transplant infectious diseases, and clinical microbiology. For over a decade, Emory University was an active member of the NIH Research Centers of Excellence (RCE) in Biodefense and was the site for the biosafety training program for select pathogens of the Southeast Regional Center of Excellence for Emerging Infections and Biodefense (SERCEB) RCE. This particular program provided invaluable expertise, equipment, and support for the EVD effort. Emory ID and Emory Healthcare (EHC) also created the TravelWell clinic, an outpatient clinic available for assessment and evaluation of pre- and post-travelers and supporting major Atlanta corporations and the CDC. Emory’s TravelWell is part of a 57-site worldwide travel network (GeoSentinel) with a 10-year database of 3,000 entries of those who either had returned ill following travel to Liberia, Sierra Leone, Guinea, or Nigeria or had just emigrated from one of those countries.

## The Decision to Accept EVD Patients into the SCDU

The request for medical care for the initial patients came from the missionary organizations Samaritan’s Purse and Serving in Mission (SIM) USA and from the patients themselves. The decision was coordinated through the US State Department and the CDC. EUH and EHC leadership made the decision to accept the patients—a decision made quickly but not lightly. The decision was based on the need, confidence in the overall preparedness of EUH, and the more than a decade plus of training and preparation to address highly communicable diseases at EUH (Box 2 and Box 3). Importantly, as an academic medical center and research institution, the decisions were also based upon what we might learn in treating these patients (e.g., advancing knowledge about human health) and how such knowledge could be applied to the current outbreak.

**Box 2. SCDU Preparation and Operations**

- The SCDU medical team is led by the SCDU director, an ID trained physician who has spearheaded for over a decade efforts in establishing the protocols, training, education, and operations of the unit in conjunction with the CDC. Three other faculty members of the Department of Medicine, four additional ID physicians, two pathologists, five medical technologists, and 21 critical care nurses also support the unit.
- An SCDU unit director, clinical nurse specialist, and team coordinator handle logistics, organize training sessions, determine supply needs, and establish nursing schedules. Nurses provide care in 4-hour stretches to minimize donning and doffing of personal protective equipment. The overall effort has directly involved over 125 staff and support personnel.
- During the care and treatment of EVD patients, there were daily communications between the SCDU medical team and the CDC’s Division of High-Consequence Pathogens and Pathology (DHCPP), the Division of Healthcare Quality Promotion, and the Emergency Operations Center for Ebola established at CDC, and with the US Food and Drug Administration (FDA) and other US federal agencies, as well as frequent communications with other academic units, industry, and providers in West Africa.
- The primary focus of training was on biosafety for all individuals who provided direct care or handled body fluids. All of these individuals received mandatory education and training, followed by strict competency verification before medical or nursing staffs were permitted to provide care for these patients.
- Effective and assertive communication was central to the safety of the SCDU team. Use of the buddy system was an essential element of the focus on individual and colleague safety. Because communication was so important, the team developed rules to govern direct patient care communication as well as within daily team huddles. These rules were reviewed each morning during the daily huddle and served as a platform to empower all members of the team, regardless of role, to facilitate communication and to maintain strict SOPs.

**Box 3. The SCDU Support Teams**

- The hospital support team was led by the CEO of EUH. The chief medical and nursing officers of EHC and EUH, and in particular the vice president of operations of EUH, were also heavily involved in leading the support efforts. During the care and treatment of the EVD patients, an operations team of key administrators and nursing and physician leadership met twice a day. Despite the extensive preplanning, opportunities to refine and improve both clinical and operations were noted, and all aspects of the hospital services were involved. Pharmacy, environmental services, engineering/facilities support, and pastoral care/counseling were especially important.
- Other ID faculty, including a pediatric ID specialist with experience in EVD, provided support for the frontline medical team. Periodic conference calls focused on support, therapeutic options in treating patients, and issues of postexposure prophylaxis, vaccines, surge planning, protocol development, and discussions of research as it related to EVD. To facilitate the necessary availability of SCDU dedicated ID providers, alternate faculty were recruited to cover the regular duties of the SCDU physicians. ID support also included contacting key national and international research leaders in EVD; evaluating, contacting, and obtaining, through outside groups, experimental vaccines and new drugs for Ebola; and communicating with the CDC, FDA, NIH, and other federal agencies, other university partners, and biotech/industry contacts, all of which were essential in the effort.
- The Emory Environmental Health and Safety Office (EHSO) was engaged immediately after the call for ongoing support. While clinical care settings are very different from those of labs, EHSO staff included individuals with expertise in training on Biosafety Level BSL 3 and 4 laboratory safety, which was helpful in translating to our clinical care situation. Conversations were conducted by EHSO with other national biosafety leaders in EVD. (See “Biosafety.”)
- The SCDU team was also supported by the Department of Emergency Medicine and the Emory Office of Critical Event Preparedness and Response focusing on the complexity of patient transport and emergency care. They worked closely before and during the event with Phoenix Air Group, the air ambulance transport service, and the Grady Hospital Ambulance service. Critical Care established a 24/7 Medical Intensive Care Unit (MICU) attending coverage for critical care management issues including airway management and line placement issues. Other subspecialties, such as renal medicine, were required on a case-by-case basis. The location of the SCDU away from other patient care units of EUH significantly reduced the impact on our other health care delivery and patient operations. DHCPP at CDC provided critical help to the unit for viral loads, specialized assays, and other support.

## Special Challenges of Addressing EVD

### Biosafety

Different outside perspectives and guidance were initially provided about the level of biosafety needed for EVD. Ebola viruses have a very low infectious dose, 1–10 aerosolized organisms, in nonhuman primate models [12] and can reach extremely high levels in blood (up to 10^10^ RNA copies per ml of serum [13]), stool, sweat, and other body secretions. Exposure of health care workers to infected body fluids via emesis, sweat, urine, saliva, diarrhea, or blood is a significant concern. There was also initial concern that this strain was not behaving epidemiologically as expected and the personal protective equipment (PPE) guidelines in Africa were not fully effective in prevention of transmission. Further, we had to demonstrate our ability to safely manage these patients.

To achieve the lowest risk to health care personnel, the highest level of biosafety was initially employed. This included MAXAIR powered air-purifying respirators (PAPRs), fluid-resistant body suits along with cover aprons, double gloves and shoe covers, and wiping down all surfaces with US Environmental Protection Agency (EPA)-registered disinfectants (no spraying) when doffing PPE. Other key biosafety SOPs developed were autoclaving within the unit for waste management, followed by disposal of regulated medical waste off-site after it had been autoclaved; decontamination; and handling of laboratory samples and waste. The consequences of transmission stressed the need for the highest level of biosafety.

The importance of the EHSO office in reinforcing the prior training of personnel and monitoring the clinical staff for consistency and adherence to biosafety from training sessions to practices in real-time clinical care cannot be overemphasized. This included how to “don and doff” impermeable fluid-resistant body suits, proper use of PAPRs, coordination of the disposal of waste, and the handling and transport of lab samples. EHSO also helped prepare and support the team psychologically, challenging them with spills and other incidents to practice remaining calm in the face of potential unexpected occurrences. Team engagement demonstrated the importance of melding the philosophies of clinical treatment/practice and biocontainment/biosafety. Feedback from frontline physicians, nurses, and hospital staff highlighted the contributions of EHSO to the effort and the value of the EHSO biodefense education and training expertise.

### Waste Management

Waste management for EVD is a significant hurdle. EVD results in copious amounts of vomit and diarrhea with up to 10 liters of fluid lost by patients each day. Up to 40 large “bags” per day of regulated medical waste were generated by our first two patients. A large-capacity autoclave was mobilized, with postautoclaved waste stored in 32-gallon leak-proof rubber waste containers in the SCDU until removal and incineration. All waste in toilets was disinfected with an EPA-registered disinfectant before flushing. Importantly, no traces of Ebola RNA on "high-touch surfaces" such as beds and bathrooms were detected during active care or after patient discharge prior to terminal decontamination with vaporized hydrogen peroxide.

### Communications

Guiding principles for communications included consistent messaging and protecting patient privacy while educating the public. Internal communications with a focus on transparency and education, within the bounds of confidentiality of medical information and protecting our patients’ privacy, were vital. These included meetings with EUH hospital leadership and staff to address concerns, frequent emails and other communications with all faculty and staff at EHC and Emory, making sure instructions/checklists were in place, and facilitating the dissemination of protocols. To avoid unauthorized access to electronic medical records, a chart-warning flag was implemented. Confidentiality of medical information regulations were repeatedly stressed to all medical staff, residents, and fellows.

In external communications and interface with the media, the Emory communications teams tirelessly addressed media logistics, developed web and social media material, worked closely with governmental and nongovernmental external groups, developed talking points, and prepared the hospital and SCDU teams for communications venues. To maximize our preparedness and to address national concerns, press conferences and media interviews were conducted, and an EVD Questions and Answers document was developed and posted. The story exploded in broadcast print and social media. Over 84,000 media stories have been written linking Emory and Ebola. Emory communications created an Emory Healthcare Ebola Preparedness Protocols website (www.emoryhealthcare.org/ebolaprep) [11] to make our clinical and biosafety protocols and other information widely available to the public. To date, 20,267 registrants have downloaded these educational materials.

### Addressing a Surge of Cases of Possible Ebola

The admissions heightened Ebola and other bio-threat concerns both locally and nationally. Questions about potential EVD patients began to occur immediately in our health care system and throughout the US. We developed and disseminated flow diagrams, algorithms, and SOPs [11] for evaluating patients in emergency rooms and other health care provider settings where EVD was a consideration. CDC guidelines as they became available and current EVD articles were also disseminated. Our teams engaged local, regional, state, and national health care groups, and guidance was provided to questions about travelers from the region coming to Emory, to airlines flying to West Africa, and to multinational corporations based in West Africa. Travelers’ diarrhea with fever and malaria are the problems most commonly encountered by travellers to West Africa. However, yellow fever along with Lassa fever, endemic in West Africa as well, can cause similar symptoms to those of Ebola. Typhoid and less frequently diseases such as dengue and other viral syndromes can cause such signs/symptoms, though not usually as severe. The intersection of the seasonal influenza epidemic with returning CDC workers from West Africa further amplified the evaluations of febrile episodes. Timely initial screening—with establishment of contact and droplet precautions early while the risk assessment, including an extensive travel history and the evaluation of exposure risk and symptoms, was continued—was emphasized. Policies and procedures for the continuing care of our discharged patients were also developed [11].

### Disseminating Knowledge: Education and Training

As noted, a highly trained group of faculty, nurses, biosafety support, and other providers staff the EUH SCDU. While no trainees (e.g., fellows, residents, or students) served on the direct care team, trainees benefitted from the experience of the unit and clinical experience with EVD through conferences and other education venues (e.g., Public Health Grand Rounds and ID seminars). An EVD “Micro Vignette” and other educational materials were developed for resident and fellow education. Further, ID and other specialty fellows have been integrated into the management planning for EVD patients, educated on the broader policies and procedures of the unit, are involved with the development of protocols for the management of Ebola patients who present to the Emergency Department (ED) or other clinical settings, and are educated about the collective experiences in treating the patients. In addition, a Massive Open Online Course (MOOC) “Ebola at Emory: Patients to Populations” has been launched [14], an EBOLA Academic Learning Community was established, and other efforts to educate and support graduate and undergraduate student activities were undertaken. Emory faculty and staff also actively participate in national education and training efforts, including multiple presentations at national meetings, engagement in training forums sponsored by the CDC and the US Department of Health and Human Services (HHS), writing publications and communications, and conducting press conferences. Fifty-five US hospitals, including many in academic medical centers, have now been designated as Ebola treatment centers.

### Generating New Knowledge

Work was initiated to better understand EVD pathogenesis and therapeutic options [7–11,15–19], including optimal patient management, fluid and electrolyte replacement, and dialysis [7–8]; evaluation of concomitant infections (e.g., malaria or Lassa fever); levels of EVD viremia, monitoring viral RNA in body sites [19] and environmental samples; tracking EVD specific immune responses [17]; evaluation of available investigational drugs and their safety, risks, and benefits; options for immune therapy (monoclonal antibodies [mAbs], convalescent sera) and available supplies; available investigational vaccines [2,18], best practices in clinical operational management; and postexposure prophylaxis. In addition, the generation of new knowledge about EVD biosafety, infection control, and waste management has resulted from this event. These activities have now expanded as a broader institutional effort that includes new drug discovery for EVD, generation of human Ebola mAbs, expanded study of EVD glycobiology, focus on biomarkers as correlates of clinical EVD outcome, work on the ecology and the emergence of EVD and other tropical-associated zoonoses, clinical trials of Ebola vaccine, and modeling the spread and control of EVD in West Africa. This work required close coordination with and the support of the Emory Institutional Review Board (IRB), collaboration with the FDA, establishment of emergency investigational new drugs (INDs), and work with drug and biologic manufacturers and with multiple other governmental agencies. These and other EVD research and scholarly projects generated by the decision continue at Emory.

The event also led to broad faculty engagement across all parts of the institution through the creation of an Emory Ebola Task Force and Ebola Faculty and Community Discussion Forum [20]. The Task Force includes leaders from across the campus and is directed at facilitating cross-cutting institutional initiatives such as EVD research, education and training activities, input into public policy issues around EVD, development of an institutional travel policy to West Africa, establishing procedures for trainee involvement in Ebola care, and the support of the humanitarian, research and control efforts in West Africa.

## Conclusions

EVD has arrived in North America and Europe and impacted us globally. Academic medical centers can be expected to be at the frontline to address EVD and other highly communicable infectious agents in the future. Our experience (Box 4) with EVD emphasized prepared infrastructure and coordination of clinical care, hospital and emergency operations, transportation, infection control, biocontainment, communications, and education and training initiatives. Unanticipated major challenges can be expected. The importance of detailed planning, preparedness, and team work across multiple academic and health care units, the emphasis on biosafety, key partnerships with environmental health and safety and institutional communications, and working closely with governmental agencies and outside collaborators to manage these threats is highlighted.

**Box 4. Key Lessons and Recommendations**

- Communicable infectious diseases will continue to be global threats to human health and have the potential to cause repeated humanitarian disasters.
- Our experience as an academic health center with EVD emphasized the importance of preparation, communications, and implementation:
  - Coordination, detailed planning, and teamwork across governmental, nongovernmental, academic, and health care units
  - Preparedness in training, biosafety, surveillance, and communications
    - Environmental Health and Safety as a key support team member
  - Evaluation of provider competency in biosafety
    - Donning and doffing of PPE
    - Waste management protocols
    - Decontamination and containment protocols
    - Specimen handling for diagnostic testing
  - An organizational structure to solve unanticipated challenges and the logistics of working closely together
  - The rapid dissemination of best practice information
  - Providing education forums and frequent educational communications (both internal and external) around disease transmission and risk
  - Consistent messaging and protecting patient privacy while educating the public
  - Expanding new knowledge about the clinical course, complications and pathogenesis, diagnosis, treatment, and prevention
  - Engagement in the broader policy issues of global health care inequities

The institutional experience, while providing the opportunity to effectively care for these individuals, has led to best practice information and the education and training of others, created key academic, governmental, and community partnerships, expanded research programs in EVD, and resulted in the development and communication of new knowledge about this emerging disease. Mobilizing our diverse academic community to work collaboratively on this global problem is also an important long-term outcome.

## Abbreviations

BSL: biosafety level
CDC: US Centers for Disease Control and Prevention
CTSA: NIH Clinical and Translational Science Awards
DHCPP: Division of High-Consequence Pathogens and Pathology
ED: Emergency Department
EHC: Emory Healthcare
EHSO: Environmental Health and Safety Office
ELWA: Eternal Love Winning Africa
EPA: US Environmental Protection Agency
EUH: Emory University Hospital
EVD: Ebola virus disease
FDA: US Food and Drug Administration
HEPA: high-efficiency particulate air
ICU: intensive care unit
HHS: US Department of Health and Human Services
ID: infectious diseases
IND: investigational new drug
IRB: Institutional Review Board
IV: intravenous
mAbs: monoclonal antibodies
MICU: medical intensive care unit
MOOC: Massive Open Online Course
NIH: National Institutes of Health
PAPR: powered air-purifying respirator
PPE: personal protective equipment
RCE: Research Centers of Excellence
SARS: severe acute respiratory syndrome
SCDU: Serious Communicable Disease Unit
SERCEB: Southeast Regional Center of Excellence for Emerging Infections and Biodefence
SIM USA: Serving in Mission USA
SOP: standard operating procedure
UHC: University Healthsystem Consortium; WHO, World Health Organization
